# Turning ingroup wounds into bonds: perceptions of gender inequalities predict attitudes toward other minorities

**DOI:** 10.3389/fpsyg.2023.1327262

**Published:** 2024-01-08

**Authors:** Stefano Ciaffoni, Monica Rubini, Silvia Moscatelli

**Affiliations:** Department of Psychology, University of Bologna, Bologna, Italy

**Keywords:** gender inequality, minority groups, attitudes, intra-minority solidarity, relative deprivation

## Abstract

Despite significant strides in reducing gender disparities over the past decades, women still face disparities in several domains. While extensive research has explored the various consequences of gender inequalities for women, this study (*N* = 493 participants) delves into a less-explored dimension, investigating whether and how perceiving gender inequalities is associated with attitudes toward minorities. Drawing on relative deprivation theory and intra-minority solidarity research, we examined the relationship between women's perceptions of gender inequalities—spanning workplace inequality, domestic inequality, sexual harassment, and social expectations—and attitudes toward gays and lesbians, transgender women, and immigrants. We also explored whether indignation, arising from recognizing unjust circumstances, mediated these relationships, and the moderating role of perceived friends' support for gender equality. The results of the path analyses unveiled a nuanced relationship. While women who were more aware of gender inequalities exhibited more positive attitudes toward gays and lesbians and transgender women, no such relationship was observed regarding immigrants. Indignation and perceived friends' support for gender equality were key factors in fostering positive intergroup attitudes. Regarding their moderating role, perceived social norms only influenced the relationship between indignation and attitudes toward gays and lesbians. These findings shed light on the intricate interplay between gender inequalities and minority group attitudes. Recognizing the multifaceted nature of gender inequality and its emotional impact can catalyze promoting coalitional attitudes and collective action among disadvantaged groups. The study also underscores the potential of close groups' norms in promoting positive intergroup attitudes, warranting further exploration.

## 1 Introduction

Even though in the last 50 years, disparities between men and women have decreased in Western societies, inequality and discrimination based on gender are still a common phenomenon (World Economic Forum, 2020; Riquelme et al., 2021). Women globally earn 20% less than men at work while carrying out at least 2.5 times more unpaid work (ILO, 2022). They also continue to be victims of discrimination in other domains: for instance, the European Union Agency for Fundamental Rights (2015) estimated that about 55% of European women were targets of unwanted sexual harassment at least once in their lifetime.

Research has pointed out how gender inequalities in the work and domestic domains restrict women's access to education, jobs, and career opportunities, and has highlighted the pervasive consequences of sexual harassment and everyday instances of gender discrimination (e.g., sexist remarks; sexual objectification) on women's wellbeing (Hackett et al., 2019; Vigod and Rochon, 2020). To our knowledge, less attention has been paid to more distal correlates of gender inequalities, such as intergroup attitudes and prejudice. Analyzing women's role within intergroup relations is vital for both advancing understanding and facilitating social justice. Constituting over half of the global population (United Nations, Department of Economic and Social Affairs, Population Division, 2022), women wield significant numerical influence that can shape social dynamics. Because of this vital importance, this study aimed to assess whether and how the perception of being the target of gender inequalities relates to women's attitudes toward other disadvantaged groups.

Based on the existing literature, two opposite patterns of relationships can be plausible. On the one hand, relative deprivation theorization would lead to expect that the perception of gender inequalities is related to greater prejudice toward minority groups (Runciman, 1966; Smith and Pettigrew, 2014). On the other hand, research on intra-minority solidarity has shown that under certain conditions, minority membership might also foster positive attitudes toward outgroups (Craig and Richeson, 2012, 2016).

The present study aimed to address this issue by examining whether and how perceiving gender inequality was related to women's attitudes toward minority groups. Acknowledging that gender inequality has a multifaced nature, we considered women's subjective perception of their disadvantaged stand along different domains and their emotional reactions to such perceptions. Given the power of social norms—that is, shared beliefs and prescriptions concerning the appropriate conduct for group members (Ajzen, 1991; Jetten et al., 1996)—as drivers of intergroup attitudes (Crandall et al., 2002), we also explored whether perceived social norms of one's group of friends, related to gender equality, worked as a moderator of the relationships under investigation. To address these aims, we focused on women's attitudes toward three minorities that in Italy, where the study was conducted, are often targets of stigmatization, such as gays and lesbians, transgender women, and immigrants (Ferrari, 2018; Valbruzzi, 2018; Federico, 2023).

### 1.1 Relative deprivation as a driver of prejudice against minorities

Perceiving that one's group is subjected to unfair treatment is a powerful psychological phenomenon. If the comparison between the conditions of the ingroup and the outgroup leads individuals to perceive that their group is not granted what it deserves, individuals are likely to experience group relative deprivation (for reviews, see Smith and Pettigrew, 2014; Anier et al., 2016). Such experience is in principle independent from one's factual situation and the objective prestige or wealth of the group itself; in fact, even members of objectively advantaged groups can feel that they are being treated worse than deserved compared to a disadvantaged outgroup (Vanneman and Pettigrew, 1972; Crosby, 1976).

Group relative deprivation has been related to a greater willingness to act for social change (Smith et al., 2012; Agostini and van Zomeren, 2021; see also Mazzuca et al., 2022) but also to more negative attitudes toward outgroups (Moscatelli et al., 2014; Anier et al., 2016). Pettigrew et al. (2008), analyzing data from different European countries, showed that the more individuals reported feelings of being relatively deprived as citizens of their countries, the more they exhibited prejudice against immigrants. A similar pattern was found in the South African context (Dambrun et al., 2006). What is interesting, is that when people experience group-based relative deprivation they do not only report more negative attitudes toward groups that are better off, threatening or somehow responsible for their group's situation (Moscatelli et al., 2014; Meuleman et al., 2020) but tend to show prejudice toward other stigmatized groups as well (Guimond and Dambrun, 2002; Eller et al., 2020; see also Jetten et al., 2015).

The main reason why perceiving that the ingroup is unfairly disadvantaged has such an impact is that it fosters the experience of the so-called justice-related emotions, such as anger, resentment, or indignation, especially if one thinks that the situation is changeable (van Zomeren et al., 2004; Smith et al., 2012). Such justice-related emotions are key in understanding the consequences of cognitive appraisals of one's group situation and were found to mediate the association of relative deprivation with collective action intentions and intergroup attitudes (Smith et al., 2012). In particular, anger and resentment are strongly associated with readiness to act (Leach et al., 2002), whereas feelings of indignation are especially likely to arise in response to perceived injustice and violation of moral values (Lazarus, 1991; Leach et al., 2007).

Whereas, relative deprivation theory has emphasized the role of justice-related appraisal and emotions, it should be noted that other psychological processes can also account for minority groups' discrimination against other minorities. System justification theory claims that people have epistemic, existential, and relational needs to justify the status quo, and one of the ways in which this occurs is by discriminating against the disadvantaged, for example by thinking that ultimately they deserve to be at the bottom of society (Jost, 2019). Moreover, people who are discriminated against, such as established immigrant communities, can discriminate against other minority groups (e.g., new immigrants) when they see such groups as a threat in the labor market (Meeusen et al., 2019) or a threat to the value of their social identity (Branscombe et al., 1999). In the latter case, according to social identity theory (Tajfel and Turner, 1979), discrimination against lower-status outgroups can represent a defensive response through which members of a disadvantaged group try to re-establish their collective self-esteem (e.g., Kessler and Mummendey, 2001).

### 1.2 Relationships between minority groups: competition or solidarity?

Albeit frequent, outgroup derogation is not the only response to the ingroup disadvantaged status (Craig and Richeson, 2012, 2016; Ball and Branscombe, 2019). For instance, when established immigrant groups see themselves as unfairly treated by the native population or the governmental institutions, a sense of commonality and empathy with minorities who share a similarly vulnerable position is likely to arise (Craig and Richeson, 2012). These feelings have been conceived as instances of intra-minority solidarity, as they arise from the assimilation of another minority's struggles as one's own, often accompanied by a moral obligation to challenge the status quo or even by active support for outgroup rights (Sirin et al., 2017; Ball and Branscombe, 2019; Meeusen et al., 2019).

In line with the social identity approach (Turner and Reynolds, 2001), intra-minority solidarity can replace outgroup discrimination if individuals come to identify with a superordinate common category that includes the former ingroup and outgroups (e.g., Gardham and Brown, 2001; Gaertner et al., 2016). Namely, the common experience of discrimination on behalf of a certain identity dimension (e.g., race) may activate a superordinate common category (e.g., “racial minorities”) and foster solidarity between minorities that pursue a common objective or connect through similar experiences of oppression (Cortland et al., 2017; Ball and Branscombe, 2019). Yet, research has also found that minority groups are still likely to derogate outgroups that are stigmatized along a different dimension (Craig and Richeson, 2016). For instance, (straight) racial minority members showed more negative attitudes toward sexual minorities after being exposed to racial discrimination against their group (Craig and Richeson, 2014). In similar cases, feelings of competitive victimhood might have been induced, so that groups compete with each other to claim the relative victim status for their ingroup (Young and Sullivan, 2016).

### 1.3 From gender inequalities to attitudes toward minorities

The literature on both relative deprivation and intra-minority solidarity has mostly focused on ethnic minorities, and women have been hardly considered (e.g., Anier et al., 2016; Craig and Richeson, 2016). As an exception, Craig et al. (2012) found that manipulated salient sexism enhanced the racial bias against Black people and Latinos in a sample of White women. Nevertheless, what remains to be clarified is whether the perception of gender inequalities—along various dimensions—relates to women's attitudes toward other minority groups.

Apparently, women do not embody the prototypical minority group within society: They are not numerically inferior to the majority (e.g., men), have—at least in principle—the same power, and do not need to claim specific rights as migrants or sexual minorities do. Nevertheless, women represent a minoritized group, as in all societies, they are by no doubt disadvantaged in multiple domains—from work, money, time, and power to health and education—and are targets of gender violence (ILO, 2022; EIGE, 2023). This disadvantage can take up very subtle forms, is oftentimes internalized and somewhat justified (e.g., Jost and Kay, 2005), and permeates every aspect of life, from intimate relationships to structural barriers to economic empowerment (e.g., Heilman, 2012; Ellemers, 2018; Alba et al., 2023).

In the attempt to capture the most salient and widespread experiences of gender inequalities in Western society—from the perspective of women—Ciaffoni et al. (2023) proposed that four forms of inequalities should be considered. First, women can perceive differences between men and women in the work domain, that is, restrictions in job and career opportunities for women, or biased expectations at work (i.e., workplace inequalities; Ryan et al., 2016; Moscatelli et al., 2020; Menegatti et al., 2021). A second, more general form of gender inequalities is represented by the prevalence of *harassment toward women*, that is, a series of subtle or more explicit undesired sexual advances, requests for sexual favors, catcalling, or other behaviors that can offend, humiliate, or intimidate women (Brown et al., 2020; WHO, 2021).

Gender inequality can also concern more private domains, less likely to be widely debated. For instance, a still prevalent form of gender inequality is represented by *domestic imbalance*, that is, an unequal distribution of domestic duties to women. This is often so deeply ingrained in society's functioning that it is not even considered unfair (Trappe et al., 2015; Cerrato and Cifre, 2018). Finally, in daily life, women face unspoken yet potent gender inequalities, such as societal pressures to meet beauty standards, be attractive to men, and prioritize motherhood (i.e., social expectations; Ashburn-Nardo, 2017; Moscatelli et al., 2021). These expectations limit women's freedom of action (e.g., Nelson and Brown, 2019; Kuipers et al., 2021).

All in all, these studies point out that understanding reactions to gender inequalities should not ignore that inequalities in different domains are likely to have different repercussions for women's lives (Ciaffoni et al., 2023). In this respect, a further critical factor is represented by one's perception that significant others justify or contrast such inequalities. Research has highlighted that people tend to adjust their views to those that are prevalent within their social groups (e.g., Crandall et al., 2002; Cialdini and Goldstein, 2004). For instance, students exposed to a message according to which their peers (the university community) valued diversity and engaged in inclusive behaviors toward people from all social backgrounds reported greater endorsement of diversity (Murrar et al., 2020). Normative influence is even higher when norms have an injunctive (i.e., they reflect what most others approve or disapprove of) rather than a descriptive function (i.e., they reflect the perception of whether other people perform a certain behavior; Smith and Louis, 2008).

According to a social identity perspective, people are more likely to conform to the perceived norms of groups they strongly identify with Abrams and Hogg (2011). However, the relevance of specific sources of normative influence will vary depending on the reference context (for instance, colleagues' norms regarding the appropriate behavior will be impactful at work but easily overcome by family norms at home; Smith and Louis, 2009) as well as individuals' age, with friends becoming more influent than family as individuals approach adolescence and youth (McDonald and Crandall, 2015; Murrar et al., 2020; Bracegirdle et al., 2022). Thus, it seems plausible that women's responses to perceived gender inequality would be influenced by the perception that their close friends hold (descriptive and injunctive) pro-gender equality norms.

## 2 The present study

As mentioned, some critical gaps in the literature can be pointed out. Relative deprivation and intra-minority solidarity traditions have paid limited attention to women as a disadvantaged group. Furthermore, studies have not considered the heterogeneity of gender inequalities and how they relate to women's intergroup attitudes. This study aims to address these issues by examining the relationships between perceptions of gender inequality—along the dimensions of workplace inequality, domestic inequality, sexual harassment, and social expectations (Ciaffoni et al., 2023) —and attitudes toward other minorities: gays and lesbians, transgender women, and immigrants. In addition, it tested whether such relationships were mediated by indignation. While recognizing that perceiving gender inequalities might trigger a wider range of emotional responses, including anger and resentment. Such emotions seem more likely to be directed against the causes or the groups responsible for the disadvantage and are known to relate to actions to improve the ingroup situation (Leach et al., 2002, 2015). Indignation represents instead a moral emotion triggered by the acknowledgment of unjust circumstances and the violation of social rules and rights, in particular the rights of others (e.g., Neblett, 1979; Hansberg, 2000). Thus, as indignation is more directedly connected to the recognition of injustice rather than to intense arousal leading to action (e.g., Lazarus, 1991; Leach et al., 2015) we reasoned that it could play a role in the relationship between the perception of one's group disadvantage and attitudes toward other disadvantaged groups.

Given that friends exert a great influence on attitudes toward outgroups (Norman et al., 2005; McDonald and Crandall, 2015), and that the inclusion of women of different ages, marital, and occupational status in our sample would have rendered it difficult to consider other types of groups (e.g., colleagues or family), in this study we explored whether women's responses to perceived gender inequality were moderated by perceived friends' norms about supporting gender equality. Since political orientation and age are generally associated with attitudes toward LGBTQ+ minorities and immigrants—with left-wing oriented and younger people being more favorable toward those groups compared to right-wing oriented and older people (Prati et al., 2018; Russo et al., 2019; Abdelaaty and Steele, 2022)—we included political orientation and age as covariates in the analyses. Finally, participants' sexual orientation was included as a covariate, since more favorable attitudes toward gay and lesbian and trans women can be expected by queer rather than heterosexual people.

The study was run in Italy, a context where stereotypic views of women and gender inequalities are quite widespread (ISTAT, 2019; Moscatelli et al., 2021; Menegatti et al., 2022; Ostuni et al., 2022). For instance, the gender employment gap reaches 20%, which is twice as high as in most European countries, and at least 21% of women undergo sexual violence in their lives (EIGE, 2015). As claimed by Galizzi et al. (2023), patriarchy, intended as male dominance, persists and permeates the Italian culture within the family and society.

In general terms, different predictions might be advanced considering the existing literature. Based on relative deprivation theory (Runciman, 1966; Smith and Pettigrew, 2014), one might expect that the more women are aware of gender inequalities and experience indignation, they would show greater prejudice against other disadvantaged and stigmatized groups. Conversely, drawing from research on intra-minority solidarity (Craig and Richeson, 2016), it is possible that women who perceive gender inequalities to a greater extent and feel greater indignation, would be more sympathetic toward other minorities and therefore report more positive attitudes toward them.

Even though this study had an explorative nature, noticing some specificities about the three outgroups considered can help advance tentative expectations. With respect to gays and lesbians, they are not necessarily stigmatized along the same identity dimension as women, but both groups suffer discrimination stemming from the endorsement of typically masculine and patriarchal views, which may make identifying shared external threats easier (Inglehart et al., 2017). Furthermore, beyond the potential overlap between the two groups (i.e., lesbian women), coalitions between activists for gender and sexual equality are common, too (Uysal et al., 2022). Intra-minority solidarity—that is, positive associations between gender inequalities and favorable attitudes toward gays and lesbians—seems therefore plausible.

Regarding transgender women, the situation is more complex. Even though both transgender and cisgender women are stigmatized along the same identity dimension, cisgender women sometimes perceive transgender women as an identity threat (Broussard and Warner, 2019). One such example is the ongoing debate around womanhood and trans women's right to access “women's spaces” (Leante, 2021; Maxwell et al., 2023). Nevertheless, the spreading of “transfeminism” —a branch of feminism that endorses the principles of intersectionality—has underlined the importance of fighting patriarchal culture and pursuing the common goal of gender equality (Bunker, 2023). Thus, despite the complexity of the positions concerning trans women, it seems plausible that a greater perception of gender inequalities would be related to more positive views of trans women.

Of the three groups, that of migrants is the one that can be seen as more distant from women, because stigmatized on a completely different dimension (race vs. gender). While gender discrimination assumes very different forms and often goes undetected (Woodzicka et al., 2015; Argüello-Gutiérrez et al., 2023), in Italy discrimination against migrants often takes quite blatant forms and translates into overt positions against migrants' rights (e.g., Fulvi, 2022). Furthermore, migrants, especially those from non-Western countries, are often depicted as promoting sexist views of women and even associated with episodes of sexual abuse of women (Belpietro, 2022). Despite the possible overlap (i.e., women migrants), it seems hard to expect intra-minority solidarity when migrants are considered as an outgroup, and the opposite pattern (that is, higher perception of gender inequalities related to less favorable attitudes toward migrants) appears more plausible. Finally, given that people tend to adjust their views to the perceived normative views of the groups they belong to (e.g., Crandall et al., 2002), one might expect that a greater perception that one's friends support gender equality would result in more positive associations between perceptions of gender inequality, indignation, and favorable attitudes toward gays, lesbians and trans women.

## 3 Materials and method

Participation in the study was completely voluntary. We recruited 657 Italian participants from the general population through personal contacts and free advertisements on social media (Facebook, Instagram, Telegram). From this initial sample, we eliminated participants who did not give their informed consent (*n* = 2) or failed to complete the central questions for this study (*n* = 151). We also excluded participants who identified as men (*n* = 2) and those who did not disclose their gender (*n* = 2). Furthermore, to ensure better-quality data, throughout the questionnaire, we added three attention checks stating “If you are paying attention, please answer strongly disagree” and we excluded those who failed more than one of three attention checks (*n* = 6). The final sample was made of 493 participants (*M*_*age*_ = 24.05, *SD* = 5.74; age ranged from 18 to 64). We decided to recruit at least 400 participants, as according to Fritz and Mackinnon (2007), these are sufficient to detect small/medium indirect effects in mediation, assuming an alpha of 0.05 and a power of 0.80. Demographic characteristics can be found in the Supplementary material - Table 1.

### 3.1 Measures

After giving informed consent, participants were presented with measures of perceptions of gender inequalities, indignation, friends' norms about supporting gender equality, and attitudes toward gays and lesbians, trans women, and migrants. Last, they reported demographic information (age, nationality, gender, sexual orientation) and political orientation. In total, the questionnaire took ~8–12 min to be completed.

Perceptions of gender inequalities were measured with the 16 items of the Multidimensional Gender Inequalities Perception Inventory (MGIPI; Ciaffoni et al., 2023). An example item is “When looking for a job, women are less likely to be hired than men.” Responses were given on a 7-point Likert scale from 1 (*strongly disagree*) to 7 (*strongly agree*). All the subscales exhibited good reliability levels (*Domestic Imbalance*, with α = 0.81, *Harassment toward Women*, with α = 0.71, *Work Inequalities*, with α = 0.80, and *Social Expectations*, with α = 0.76).

Participants' level of indignation was measured by asking “When thinking about inequalities between men and women, how much indignation do you feel?” (1 = *not at all*; 7 = *very much*; Ciaffoni et al., 2023). To measure close friends' perceived social norms we included five *ad hoc* items assessing descriptive and injunctive norms around supporting gender equality (α = 0.75). Two example items are “My closest friends support gender equality” and “My closest friends would approve if I supported pink quotas” (1 = *not at all*; 7 = *very much*).

Attitudes toward gay people were measured with the Attitudes toward Homosexuality Scale (Anderson et al., 2018), containing 16 items such as “Gay people disgust me” (α = 0.91). Attitudes toward trans women were measured with the relevant subscale of the Attitudes toward Transgender Men and Women scale (ATTMW; Billard, 2018), including 12 items such as “Transgender women are defying nature” (α = 0.96). As in the original paper, the items followed a definition of “transgender women”. For these two indexes, responses were given on a 7-point Likert scale, from 1 (*strongly disagree*) to 7 (*strongly agree*). Attitudes toward migrants in Italy (α = 0.96) were assessed by asking participants how favorable they were toward migrants from Eastern Europe, North Africa, Central Africa, Asia, and Latin America on a scale from 0 (*not at all favorable*) to 10 (*completely favorable*), like in Dambrun et al. (2006). Finally, participants had to indicate their political orientation on a slider from 0 (*close to left-wing ideas*) to 100 (*close to right-wing ideas*), a measure that is becoming rather common in social psychology and has the advantage of allowing participants to indicate their orientation on a continuous rather than discrete scale (Castelli et al., 2022; Cervone et al., 2023).

## 4 Results

### 4.1 Descriptive statistics and confirmatory factor analysis

All descriptive statistics and correlations among measures are presented in Table 1.

**Table 1:** Descriptive statistics and correlations among all variables.

		**Mean**	**SD**	**1**	**2**	**3**	**4**	**5**	**6**	**7**	**8**	**9**	**10**	**11**	**12**
1	Work inequalities	5.83	0.95												
2	Domestic imbalance	5.71	1.11	0.36^**^											
3	Harassment toward women	6.37	0.70	0.59^**^	0.43^**^										
4	Social Expectations	5.28	1.08	0.56^**^	0.32^**^	0.60^**^									
5	Indignation	5.86	1.30	0.36^**^	0.14^**^	0.33^**^	0.26^**^								
6	Perceived social norms	5.63	0.91	0.05	−0.06	0.134^**^	0.09	0.02							
7	Attitudes toward homosexuality	6.33	0.73	0.26^**^	0.03	0.31^**^	0.35^**^	0.28^**^	0.33^**^						
8	Attitudes toward trans women	6.15	1.10	0.32^**^	0.04	0.30^**^	0.34^**^	0.25^**^	0.27^**^	0.80^**^					
9	Attitudes toward Migrants	8.61	1.93	0.16^**^	0.01	0.14^**^	0.16^**^	0.15^**^	0.22^**^	0.49^**^	0.52^**^				
10	Age	24.05	5.74	−0.09	−0.06	−0.27^**^	−0.19^**^	0.01	−0.06	−0.22^**^	−0.16^**^	−0.13^**^			
11	Sexual orientation (dummy)	–	–	0.04	−0.02	0.06	0.13^**^	−0.01	0.06	0.24^**^	0.20^**^	0.14^**^	−0.13^**^		
12	Political orientation	29.47	21.97	−0.24^**^	0.01	−0.13^**^	−0.20^**^	−0.13^**^	−0.16^**^	−0.45^**^	−0.48^**^	−0.37^**^	0.02	−0.23^**^	

^**^Correlation is significant at the 0.01 level (2-tailed). ^*^Correlation is significant at the 0.05 level (2-tailed).

Before assessing the moderated mediation models, we run confirmatory factor analysis for each measure (except the single-item measure of indignation and the demographic covariates). All the analyses were conducted in Mplus 8.3 (Muthén and Muthén, 2019) using the Maximum Likelihood with Robust standard errors (MLR) estimator (Satorra and Bentler, 2001). In evaluating the goodness of fit for the CFA and the main analyses, we considered several indices (Schumacker and Lomax, 2010): CFI and TLI, with values exceeding 0.90 signifying acceptable fit and values above 0.95 suggest excellent; SRMR, for which values lower than 0.8 indicate a good fit (Hu and Bentler, 1999); RMSEA, where values below 0.05 denote excellent fit (Byrne, 2011). We also inspected the 90% confidence interval of the RMSEA: when the upper bound of this confidence interval is ≤ 0.10, the model fit can be considered acceptable (Chen et al., 2008).

Considering the CFA, all the fit indices were acceptable for all measures, except for the CFI and TFI of the Attitudes Toward Homosexuality scale which were slightly below the cutoff of 0.09 (0.86 and 0.84, respectively; see Supplementary material - Table 2). Since the validity of the scale has been established in various contexts (Anderson et al., 2018; Falomir-Pichastor et al., 2019; Valsecchi et al., 2022), and the RMSEA and SRMR index were acceptable, we reasoned that these minor deviations from the cutoff values do not pose a significant threat to its reliability.

### 4.2 Path analyses

**Figure 1:**
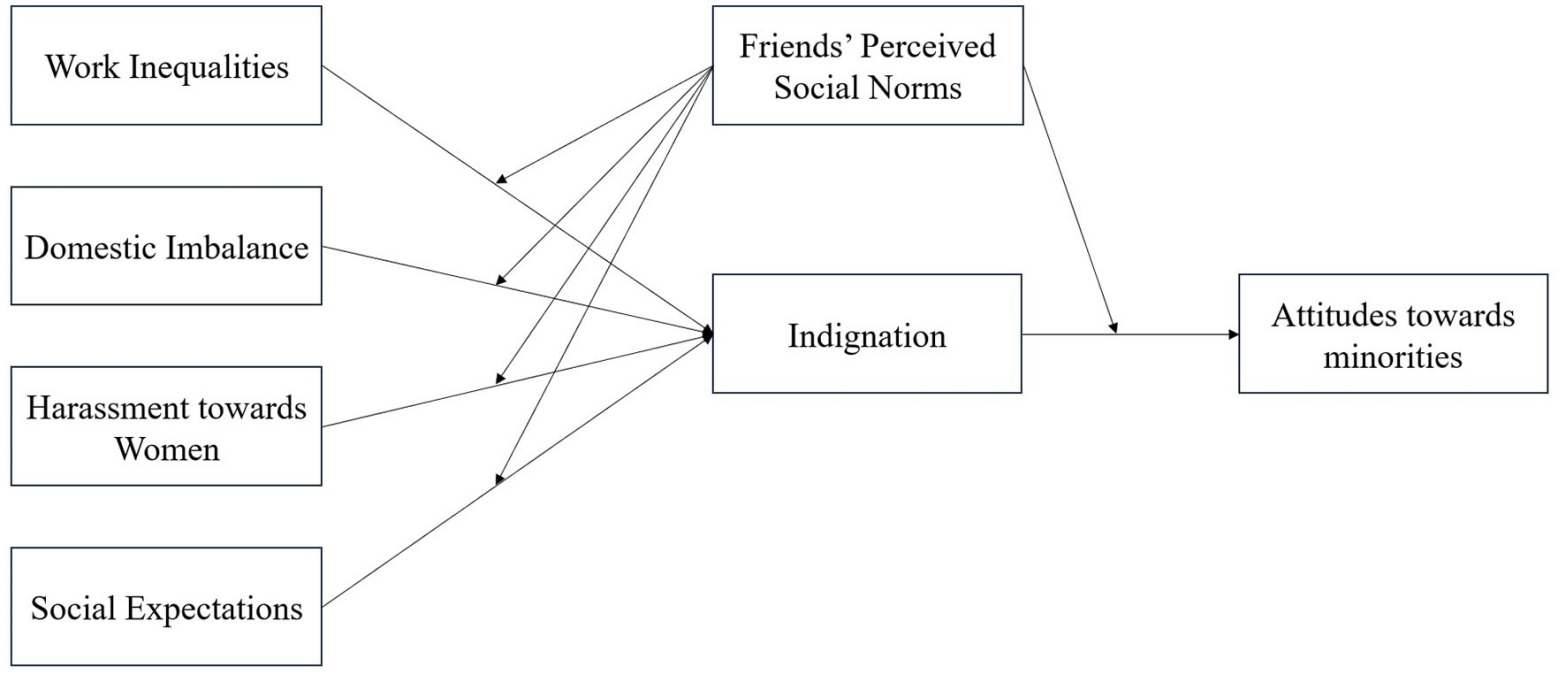
Schematic representation of the path analysis model for attitudes toward minorities.

For the main analyses, we estimated the same path analysis model on each measure of attitudes toward a minority group. The four components of perceptions of gender inequalities were included as predictors and correlated with each other (in line with Ciaffoni et al., 2023). Indignation was entered as a mediator. Perceived social norms were entered as a potential moderator of the relationships between perceptions of gender inequalities and indignation, as well as the relationship between indignation and each outcome variable (see Figure 1). Furthermore, political orientation, age and whether respondents self-identified as straight or queer were added as covariates. All variables were observed variables. The variables defining the interaction terms were centered around their mean. All the fit indices of the three models tested in this research are summarized in Table 2.

**Table 2:** Fit indices for the three models being tested in this research.

	**RMSEA**	**90% CI**	**CFI**	**TLI**	**SRMR**
Attitudes toward homosexuality	0.026	(0.000, 0.045)	0.982	0.960	0.048
Attitudes toward trans women	0.026	(0.000, 0.044)	0.982	0.960	0.048
Attitudes toward migrants	0.024	(0.000, 0.043)	0.982	0.959	0.048

Values of RMSEA and its 90% confidence interval, CFI, TLI, and SRMR for each model tested in this study.

Given that we tested three models that differed in the outcome variable only, the paths from the covariates to the predictors, the moderator, and the mediator, as well as the paths between the predictors and the mediator remained consistent across the three models and are reported in Table 3. Only perceptions of workplace inequalities and harassment toward women turned out to be significantly related to the proposed mediator: the more participants perceived gender inequalities in these two domains, the more indignation they experienced when thinking about gender inequalities.

**Table 3:** Associations between covariates, predictors, and mediator.

**Effect**	**Estimate**	***SE***	**95% CI**		***p***
			***LL***	***UL***	
**Indignation**					
**Work inequalities**	0.336	0.084	0.171	0.500	0.000
Domestic imbalance	−0.062	0.055	−0.170	0.046	0.258
**Harassment toward women**	0.428	0.129	0.175	0.680	0.001
Social expectations	0.014	0.077	−0.138	0.165	0.861
Perceived social norms	−0.050	0.063	−0.174	0.073	0.423
Work ineq. × perceived social norms	−0.001	0.081	−0.110	0.108	0.987
Domestic imb. × perceived social norms	0.010	0.064	−0.084	0.098	0.880
Harassment × perceived social norms	0.097	0.129	−0.070	0.157	0.452
Expectations × perceived social norms	0.018	0.074	−0.091	0.118	0.802
Political orientation	−0.003	0.002	−0.008	0.001	0.182
Age	0.018	0.010	−0.002	0.038	0.077
Queer vs. straight^a^	−0.095	0.157	−0.403	0.213	0.545
**Perceived social norms**					
**Political orientation**	−0.006	0.002	−0.010	−0.003	0.001
Age	−0.009	0.008	−0.024	0.006	0.247
Queer vs. straight^a^	0.081	0.100	−0.116	0.278	0.421
**Work inequalities**					
**Political orientation**	−0.010	0.002	−0.015	−0.006	0.000
Age	−0.014	0.010	−0.034	0.005	0.151
Queer vs. straight^a^	−0.082	0.114	−0.306	0.142	0.472
**Domestic imbalance**					
Political orientation	0.000	0.002	−0.006	0.005	0.917
Age	−0.013	0.010	−0.032	0.006	0.173
Queer vs straight^a^	−0.060	0.150	−0.354	0.234	0.690
**Harassment toward women**					
**Political orientation**	−0.004	0.002	−0.007	−0.001	0.013
**Age**	−0.033	0.007	−0.047	−0.019	0.000
Queer vs. straight^a^	−0.030	0.088	−0.203	0.143	0.734
**Social expectations**					
**Political orientation**	−0.009	0.002	−0.014	−0.004	0.000
**Age**	−0.033	0.011	−0.054	−0.012	0.002
**Queer vs. straight** ^**a**^	0.233	0.114	0.009	0.456	0.041

Estimates, standard errors, confidence intervals and *p*-values for each effect.

CI, confidence interval; *LL*, lower limit; *UL*, upper limit.

^a^0, straight; 1, queer.

Bolded variables are the significant predictors.

#### 4.2.1 Attitudes toward gays and lesbians

The model on attitudes toward gays and lesbians explained 42.30% of the total variance (*R*^2^ = 0.42). Of the four components of perceptions of gender inequalities, only the social expectations component had a significant and positive direct association with attitudes toward gays and lesbians (Table 4). Indignation was also positively associated with attitudes toward gays and lesbians and worked as a mediator of perceived workplace inequalities and harassment toward women, as proved by the two positive indirect effects.

**Table 4:** Direct and indirect effects on attitudes toward homosexuality.

**Effect**	**Estimate**	***SE***	**95% CI**		***p***
			***LL***	***UL***	
**Direct effects**					
**Attitudes toward homosexuality**					
Work inequalities	−0.009	0.049	−0.106	0.087	0.847
Domestic imbalance	−0.037	0.027	−0.091	0.017	0.181
Harassment toward women	0.071	0.064	−0.054	0.197	0.263
**Social expectations**	0.103	0.036	0.032	0.174	0.005
**Indignation**	0.110	0.027	0.057	0.164	0.000
**Perceived social norms**	0.204	0.032	0.141	0.267	0.000
**Indignation** **×perceived social norms**	−0.091	0.031	−0.153	−0.030	0.003
**Political orientation**	−0.010	0.002	−0.013	−0.007	0.000
**Age**	−0.019	0.006	−0.030	−0.007	0.001
**Queer vs. straight** ^**a**^	0.231	0.053	0.126	0.335	0.000
**Indirect effects**					
**Work ineq**. **>** **indignation** **>** **attitudes**	0.037	0.013	0.012	0.062	0.004
Domestic imb. > indignation > attitudes	−0.007	0.006	−0.019	0.005	0.273
**Harassment** **>** **indignation** **>** **attitudes**	0.047	0.019	0.010	0.084	0.012
Expectations > indignation > attitudes	0.001	0.008	−0.015	0.018	0.860

Estimates, standard errors, confidence intervals and *p*-values for each effect.

CI, confidence interval; *LL*, lower limit; *UL*, upper limit.

^a^0, straight; 1, queer.

Bolded variables are the significant predictors.

Perceiving that one's group of friends support equality was positively associated with favorable attitudes toward gays and lesbians, and moderated the relationship between indignation and attitudes, as shown by the significant interaction between indignation and perceived social norms. When indignation was low, participants who perceived that their friends supported gender equality showed more favorable attitudes toward gays and lesbians than participants who perceived lower support from their friends (see Supplementary material - Figure 1). Finally, all three covariates considered in the model were significantly associated with the outcome variable, so that left-wing, younger, and queer participants exhibited more favorable attitudes toward gays and lesbians.

#### 4.2.2 Attitudes toward trans women

The model assessing attitudes toward trans women accounted for 36.90% of the total variability (*R*^2^ = 0.37). Among the four components gauging perceptions of gender inequalities, only that of social expectations had a significant and positive direct association with attitudes toward trans women (Table 5). Indignation was positively linked to the outcome variable, and, in line with the previous model, we observed positive indirect effects of perception of workplace inequalities and harassment against women through indignation.

**Table 5:** Direct and indirect effects on attitudes toward trans women.

**Effect**	**Estimate**	***SE***	**95% CI**		***p***
			***LL***	***UL***	
**Direct effects**					
**Attitudes toward trans women**					
Work inequalities	0.094	0.074	−0.051	0.238	0.205
Domestic imbalance	−0.063	0.045	−0.151	0.025	0.159
Harassment toward women	0.094	0.087	−0.077	0.266	0.281
**Social expectations**	0.129	0.057	0.018	0.241	0.022
**Indignation**	0.115	0.037	0.043	0.187	0.002
**Perceived social norms**	0.220	0.046	0.131	0.309	0.000
Indignation × perceived social norms	−0.078	0.040	−0.155	0.000	0.049
**Political orientation**	−0.018	0.002	−0.022	−0.013	0.000
**Age**	−0.016	0.007	−0.030	−0.002	0.022
**Queer vs. straight** ^**a**^	0.243	0.082	0.083	0.404	0.003
**Indirect effects**					
**Work ineq**. **>** **indignation** **>** **attitudes**	0.039	0.016	0.008	0.070	0.015
Domestic imb. > indignation > attitudes	−0.007	0.007	−0.021	0.006	0.299
**Harassment** **>** **indignation** **>** **attitudes**	0.049	0.022	0.006	0.092	0.025
Expectations > indignation > attitudes	0.002	0.009	−0.016	0.019	0.860

Estimates, standard errors, confidence intervals and *p*-values for each effect.

CI, confidence interval; *LL*, lower limit; *UL*, upper limit.

^a^0, straight; 1, queer.

Bolded variables are the significant predictors.

The perception of friends' social norms in favor of gender equality was also positively related to attitudes toward trans women. Although the interaction term appears to be significant according to the *p*-value indication, it was not significant when considering the confidence interval. For the sake of thoroughness, the pattern seems aligned with what was found in the previous model: Participants who experienced low indignation showed more favorable attitudes toward trans women when they reported a higher perception of social norms in favor of gender equality (see Supplementary material - Figure 2). Furthermore, all three covariates displayed significant associations with the outcome, indicating that individuals identifying as left-wing, younger, and queer tended to hold more positive attitudes toward trans women.

#### 4.2.3 Attitudes toward migrants

The model analyzing attitudes toward migrants indicated that 19.60% of the overall variability was accounted for (*R*^2^ = 0.20). None of the four components evaluating perceptions of gender inequalities was significantly related to attitudes toward migrants (Table 6). Yet, feelings of indignation and perceived social norms were positively associated with attitudes toward migrants. No indirect effects were found to be significant.

**Table 6:** Direct and indirect effects on attitudes toward migrants.

**Effect**	**Estimate**	***SE***	**95% CI**		***p***
			***LL***	***UL***	
**Direct effects**					
**Attitudes toward migrants**					
Work inequalities	0.032	0.151	−0.264	0.328	0.833
Domestic imbalance	−0.011	0.084	−0.174	0.153	0.898
Harassment toward women	−0.032	0.170	−0.365	0.300	0.849
Social expectations	0.063	0.097	−0.127	0.252	0.517
**Indignation**	0.148	0.074	0.003	0.292	0.045
**Perceived social norms**	0.341	0.100	0.145	0.538	0.001
Indignation × perceived social norms	−0.117	0.088	−0.291	0.056	0.184
**Political orientation**	−0.027	0.004	−0.036	−0.018	0.000
**Age**	−0.035	0.017	−0.068	−0.002	0.037
Queer vs. straight^a^	0.186	0.188	−0.183	0.554	0.323
**Indirect effects**					
Work ineq. > indignation > attitudes	0.050	0.028	−0.006	0.105	0.079
Domestic imb. > indignation > attitudes	−0.009	0.010	−0.028	0.010	0.340
Harassment > indignation > attitudes	0.063	0.038	−0.012	0.138	0.100
Expectations > indignation > attitudes	0.002	0.011	−0.020	0.024	0.858

Estimates, standard errors, confidence intervals and *p*-values for each effect.

CI, confidence interval; *LL*, lower limit; *UL*, upper limit.

^a^0, straight; 1, queer.

Bolded variables are the significant predictors.

Furthermore, of the three covariates added to the model, only political orientation and age showed significant associations with the outcome variable. Individuals who identified as left-wing or were younger hold more positive attitudes toward migrants.

## 5 Discussion

The present study aimed to examine whether and how women's perceptions of gender inequalities were related to attitudes toward other minority groups, that is, gays and lesbians, transgender women, and migrants. Doing this, it bridged critical gaps in the literature, in that it considered a group that is relatively underrepresented in research concerning minority groups and delved into women's responses to the multifaced experience of gender inequalities. Moreover, albeit explorative, this study allowed us to test different predictions that can be drawn based on different theoretical frameworks, in particular, relative deprivation theory (Runciman, 1966; Smith et al., 2012) and intra-minority solidarity research (Craig and Richeson, 2016).

Overall, the findings pointed out positive associations between the perception of gender inequalities and favorable attitudes toward two of the groups considered, that is, gay people and trans women, revealing the prevalence of intra-minority solidarity. Such a logic, however, does not extend to all minorities, as suggested by the lack of significant relationships between the perception of gender inequalities and attitudes toward migrants.

### 5.1 Perceptions of gender inequalities and intra-minority solidarity

As mentioned, women who perceived greater gender inequalities reported more favorable attitudes toward gays, lesbians and trans women. The perception that women are targets of gendered social expectations had a direct association with such outcomes, whereas the findings revealed indirect effects for workplace inequality and harassment toward women. Namely, recognizing gender inequalities along such dimensions enhanced women's experience of the moral emotion of indignation, which in turn accounted for the increased positivity toward gays, lesbians and trans women. Overall, these findings are consistent with patterns of intra-minority solidarity (Craig and Richeson, 2016): the more women are aware of being subjected to inequalities—as a group—the more they show positive views of gay people and trans women.

However, the findings showed no direct or indirect links between perception of gender inequalities and attitudes toward migrants, which were instead positively associated with indignation. This finding suggests that emotional responses to inequalities, *per se*, might play a critical role that can be (at least partially) independent from the cognitive appraisal of women's conditions and may represent a critical step in fostering positive intergroup attitudes within minorities.

In an exploratory manner, our study also examined whether friends' norms regarding support for gender equality acted as a moderator of the relationships between perceptions of gender inequality, indignation, and attitudes toward minority groups. The findings only revealed some evidence of moderation with respect to the link between indignation and attitudes toward gays and lesbians, suggesting that the perception that close others support equality somehow compensates individual's low feeling of indignation for inequalities. It is also interesting that perceived social norms were directly related to more favorable attitudes toward all the groups considered. This finding suggests that being a member of a close group that supports (gender) equality might translate into more favorable attitudes toward a variety of different actions, including those aimed at improving other minorities' positions. While we are aware that more evidence is needed to support the latter contention, we believe that the role of close groups' norms deserves more attention to elucidate the conditions underlying intra-minority solidarity.

Finally, in our study, all models considered the same set of covariates, which included age, political orientation, and participants' sexual orientation. Age and political orientation emerged as significant predictors for each of our measured outcomes, with younger participants and those who identified as left-wing politically reporting more favorable attitudes toward the three minorities considered. Additionally, queer respondents showed more favorable attitudes toward gay people and trans women. These findings align with previous evidence collected in Italy and other contexts (Prati et al., 2018; Russo et al., 2019; Abdelaaty and Steele, 2022; Maratia et al., 2023).

Moreover, looking at the findings from a social identity complexity (SIC) perspective (Roccas and Brewer, 2002), one may wonder whether the different patterns we observed for migrants reflect the establishing of a unique intersection of identities, which leads to more positive attitudes toward specific minority groups (namely, gays and lesbians, and trans women) while not extending inclusivity to migrants. Future research could explore the intricate organization of these identities and how this organization influences varying degrees of acceptance of other minorities within the context of intergroup attitudes.

### 5.2 Theoretical and practical implications

Theoretically, these findings contribute to the understanding of women's experience of gender inequalities and their correlates. Perceiving gender inequalities is not only related to reduced wellbeing or higher support for gender equality actions (Davis and Robinson, 1991; Kinias and Kim, 2012) but it is also associated with more positive attitudes toward sexual and gender minorities.

These findings also add to previous studies on intra-minority solidarity (Craig and Richeson, 2016). Whereas, previous studies in this field found that women exposed to manipulated sexism showed more racial and antigay bias (Craig et al., 2012), the current findings highlighted that women's awareness of gender inequalities is positively related to attitudes toward gay people and transgender women (but not toward migrants). Such a discrepancy might be due to the lower threat that women possibly experienced in this study compared to that of Craig et al. (2012), where sexism was purposely made salient, or, alternatively, to the fact that we led respondents to focus on the variety of forms that gender inequalities can take. Thinking of the different facets of discrimination against women might have led respondents to be more empathetic toward other stigmatized groups and more prone to recognize that they, too, are discriminated against along various dimensions, thus avoiding defensive reactions and feelings of competitive victimhood (Noor et al., 2012).

Concerning the last point, it is important to underline that attitudes toward minorities (which constitute our outcome measures) can be conceived as an aspect of intra-minority solidarity, which nevertheless constitutes a more complex concept (e.g., Burson and Godfrey, 2020). Solidarity within intra-minority contexts can involve—besides attitudes and liking—support for outgroup rights (Cortland et al., 2017) or endorsement of collaborative efforts or political action on behalf of an outgroup (Glasford and Calcagno, 2012). While we believe that research on intra-minority solidarity can offer a lens through which to look at the phenomenon examined in the current study, future research should test whether perceiving gender inequalities actually translate into concrete alliance with minority outgroups (e.g., actions supporting LGBTQ+ rights).

One more consideration and possible explanation of the observed results resides in the perceived commonalities with the considered groups. Reflecting upon the different facets of gender inequalities, women might have found it easier to divert the focus from their specific condition and bring their attention to the similarities with the situation of gays, lesbians and trans women, rather than with that of migrants. Despite the specificities of the societal treatment toward those groups, cisgender women, gay people, and trans women are all targets of threats and discrimination that stem from a patriarchal culture (Valdes, 1996; Uysal et al., 2022). Theoretically, these findings seem, therefore, in line with a common identity model framework (Dovidio et al., 2007), according to which if members of different groups are induced to conceive themselves as parts of a single superordinate group, ingroup favoritism will be directed toward the new, more inclusive ingroup and therefore results in more positive attitudes toward the former outgroup. Such a theoretical model is consistent with previous evidence on intra-minority solidarity (Craig and Richeson, 2012; Cortland et al., 2017) and with the contention that, in the present study, making salient gender inequalities might have elicited recategorization processes and led women to feel as part of a more inclusive ingroup including gay people and trans women and characterized by a shared fate of discrimination by the majority group of cisgender, heterosexual men.

As a further support for such a contention, our findings revealed that perceiving gender inequalities in the domain of social expectations, workplace, and harassment toward women was related to positive attitudes toward gay people and trans women—possibly because women can easily imagine that members of such groups are targets of similar treatment as women along these dimensions. Domestic imbalance, a form of inequality that affects women but not necessarily sexual and gender minorities, was unrelated to attitudes toward gay people and trans women, and, interestingly, was not even associated with indignation, possibly because asymmetries in the domestic load are so deeply embedded in feminine norms that they do not arouse strong emotional responses in women (e.g., Cerrato and Cifre, 2018). Thus, these findings are in line with previous evidence that relating to another minority's type of oppression can facilitate solidarity (Cortland et al., 2017; Burson and Godfrey, 2020) and highlight the importance of having a nuanced look at structural inequality and consider the different ways by which structural inequality reproduces itself.

Finally, these findings also speak to the literature on relative deprivation. As discussed before, based on relative deprivation theory (Runciman, 1966), one should have expected women's perception of gender inequalities to be related to *less* positive attitudes toward other minority groups. Whereas, there was evidence of intra-minority solidarity toward sexual and gender minorities, the lack of correlations between perception of gender inequalities and attitudes toward migrants—as well as the significant association between indignation and favorable attitudes toward them—do not align with previous evidence on patterns of relative deprivation and intergroup hostility. However, it should be noted that the construct of perception of gender inequalities does not exactly coincide with that of relative deprivation (Smith et al., 2012). In fact, in the current study, the focus was on the cognitive awareness of inequalities, whereas we did not measure how legitimate they were considered (a critical aspect of the relative deprivation construct). Of course, further studies addressing the distinct role of perception (in terms of mere recognition), justice-related considerations, and emotional reaction would help clarify women's responses to gender inequalities.

Overall, these findings pave the way for interventions aimed at improving minority groups' conditions. Women often fail to recognize sexism and gender inequalities (Becker, 2010; Radke et al., 2016), and, even when they do, injunctive feminine norms of kindness and modesty make it hard to express group-based anger against inequalities (Mahalik et al., 2005). Based on these findings, one might claim that raising women's or other minority members' awareness of inequalities can help them reflect upon others' situations and can represent a first step toward the promotion of coalitional attitudes (for a similar reasoning, see Craig and Richeson, 2016). Within contexts where multiple groups grapple with the dominance of a specific culture (usually White, patriarchal, ableist, and heteronormative, at least in Western countries; Goodley, 2014), forging coalitions emerges as one of the most promising avenues to progress and achieve lasting social change. Whereas, our results can only suggest possible factors that are likely to favor such outcomes—above all, perceived intergroup similarities and common threats—professionals should be made aware of the potential of interventions based on raising the awareness of one's and other groups' situations.

In this regard, valuable insights can be gleaned from the experience of the LGBTQ+ community—whereby the common denominator is the significant social rejection members experience for belonging to gender and sexual minorities—and its successful efforts to come together with the disability community by prompting introspection regarding the shared experiences of feeling marginalized and rejected by society (Patterson et al., 2015; Ball and Branscombe, 2019). Similarly, strategic allyships between feminists and activists for LGBTQ+ rights can derive from the awareness of a common threat and the recognition of shared advantages in cooperating for social change (Acar and Ulug, 2016; Uysal et al., 2022).

### 5.3 Limitations and future directions

The study comes with several limitations. First, by relying on cross-sectional data, we can only make limited inferences about the relationships among the variables, which need to be explored further by implementing longitudinal or experimental designs. Moreover, this study did not measure whether women felt a common fate or shared goals with sexual and gender minorities. To support our interpretation of the present results in terms of intra-minority solidarity, future studies should examine whether positive attitudes toward other minority groups translate into active cooperation or actions in favor of those groups (Burson and Godfrey, 2020). Moreover, they should explore the role of possible intervening variables, such as recategorization processes, empathy and/or the identification of shared threats.

In Italy, where the study was conducted, traditional gender stereotypes and patriarchy are still pervasive (e.g., ISTAT, 2019; Pagliaro et al., 2020; Mazzuca et al., 2022). In such a context, it seems likely that women who are aware of male domination—as most women in our sample—might easily identify patriarchal culture as a critical threat to them as well as to other groups accused of undermining traditional values or who openly fight against patriarchy, such as LGBTQIA+ people. Such feelings of shared fate and common threat can explain why perceiving higher levels of gender inequalities was accompanied by more favorable views toward gay people and transgender women but not immigrant people, to which such feelings of shared destiny most likely do not apply. It is, therefore, crucial that future research clarify the conditions under which the awareness of being a disadvantaged group may result in more positive or vice versa discriminatory attitudes and behaviors toward other minorities.

Future studies should also provide more evidence on the role of perceived social norms in favor of greater equality for one's group in promoting more positive attitudes toward other minorities, hopefully leading to a greater willingness to cooperate. In a related way, it would be important to explore individuals' motivation to adhere to social norms and take a more nuanced view of such norms to delve more in-depth into their influence on women's attitudes. First, it would be interesting to understand whether women are more willing to adhere to norms endorsed by male or female friends. In the former case, one might speculate that, even in that case, women are somehow subjected to men's dominance; at the same time, such a result would prove the importance of the male alliance in fighting gender inequalities (Subašić et al., 2018). Moreover, future studies might focus on different sources of normative influence (e.g., Smith and Louis, 2009) and consider groups that might be especially relevant with respect to specific dimensions of gender inequality. For instance, perceived family norms might play a key role in supporting gender parity in the domestic sphere, whereas the perception that one's colleagues support gender parity at work might be critical when women focus on work-related inequalities.

As mentioned, the findings revealed significant associations between the three covariates we considered (i.e., political orientation, age, and sexual orientation) and attitudes toward the three minority groups, as could be expected based on previous literature (e.g., Prati et al., 2018; Russo et al., 2019; Salvati et al., 2023). Even though the analyses revealed significant effects beyond what could be attributed to these covariates, it is important to acknowledge that our sample was characterized by a predominantly young, left-leaning demographic, with a relatively high proportion of LGBTQIA+ individuals (16.5%). Thus, future studies should try to reach a more balanced and representative sample. Related to this, it is also important to recognize that by referring to women as a category, we by no means intended to deny that other categories are likely to intersect with gender and define unique experiences of discrimination and disadvantage. For instance, as pointed out by the “intersectionality” framework (Greenwood, 2008; Shields, 2008), we should keep in mind that women of other ethnic groups or women with a disability can experience violence or social expectations differently from white or women without a disability. In more general terms, even though the field has not fully come up with methodological answers to acknowledge intersectionality, we know that experiences of inequality are no one-size-fits-all phenomena, and multiple social identities (gender, ethnicity, sexual orientation, socioeconomic status, …) can overlap to shape qualitatively and quantitively different experiences of inequality.

## 6 Conclusion

Our study offers insights into the understanding of women's experience as a disadvantaged, minoritized group within society. The examination of multiple dimensions of gender inequality enriches our understanding of how gender inequalities intersect and relate to attitudes toward other minority groups. This nuanced approach highlights the complexity of the interplay between different forms of gender inequality and intergroup attitudes. Since recognizing gender inequalities, and the emotional response they raise, seems to be accompanied by higher sensitivity toward other minorities (at least, sexual and gender minorities), this study highlights the importance of interventions that increase individuals' awareness of their group's disadvantaged position while fostering contemplation on the oppression experienced by other minority groups. Leveraging the current findings to develop concrete interventions holds great potential for policymakers and activists dedicated to driving social change.

## Data availability statement

The datasets presented in this study can be found in online repositories. The names of the repository/repositories and accession number(s) can be found below: https://osf.io/zvn4h/?view_only=df350f31b113470096b483db3efa4690.

## Ethics statement

The studies involving humans were approved by Bioethical Committee of the University of Bologna. The studies were conducted in accordance with the local legislation and institutional requirements. The participants provided their written informed consent to participate in this study.

## Author contributions

SC: Conceptualization, Data curation, Formal analysis, Writing – original draft, Writing – review & editing. MR: Funding acquisition, Writing – review & editing. SM: Conceptualization, Supervision, Writing – review & editing.
