# Supplementary material for: Turning ingroup wounds into bonds: perceptions of gender inequalities predict attitudes toward other minorities
Source: Front Psychol. 2024 Jan 8;14:1327262. doi: 10.3389/fpsyg.2023.1327262 (PMC10800884; doi:10.3389/fpsyg.2023.1327262)
Supplement: Supplementary file 1 [file Data_Sheet_1.pdf]

# *Supplementary Material*

**Table 1. Sociodemographic characteristics of the participants**

| Demographic characteristic | *N*=493 | |
|---|---|---|
| | *n* | % |
| **Gender** | | |
| Women | 489 | 99.20 |
| Men | - | - |
| Non-binary | 4 | .80 |
| **Nationality** | | |
| Italian | 466 | 94.60 |
| Portuguese | 1 | .20 |
| Moldavian | 1 | .20 |
| Brazilian | 1 | .20 |
| Albanian | 4 | .80 |
| Indian | 1 | .20 |
| Bulgarian | 1 | .20 |
| Tunisian | 2 | .40 |
| Romanian | 1 | .20 |
| Swiss | 1 | .20 |
| No answer at all | 14 | 2.80 |
| **Sexual orientation** | | |
| Straight | 396 | 80.40 |
| Bisexual | 45 | 9.10 |
| Homosexual | 8 | 1.60 |
| Pansexual | 16 | 3.20 |
| Asexual | 4 | .80 |
| Queer | 3 | .60 |

| | | |
|---|---|---|
| Other | 6 | 1.20 |
| Preferred not to say | 8 | 1.60 |
| No answer at all | 7 | 1.50 |
| **Educational level** | | |
| High school | 247 | 50.20 |
| Bachelor's degree | 176 | 35.80 |
| Master's degree | 54 | 10.8 |
| Second Level Master | 1 | .20 |
| PhD | 4 | .80 |
| Other types of education | 1 | .20 |
| No answer at all | 10 | 2.00 |
| **Employment** | | |
| Unemployed | 2 | .40 |
| Student | 108 | 21.90 |
| Employed | 345 | 70.0 |
| Employed and student | 22 | 4.50 |
| Looking for 1st occupation | 3 | .60 |
| Housewife | 4 | .80 |
| Retired | 1 | .20 |
| Preferred not to say | 1 | .20 |
| No answer at all | 7 | 1.40 |

*Note.* Frequencies and percentages describing the demographic characteristics of the sample.

**Table 2. Results of the Confirmatory Factor Analyses for the utilised measures.**

| | RMSEA | 90% CI | CFI | TLI | SRMR |
|---|---|---|---|---|---|
| Perceptions of gender inequalities | 0.039 | [0.029, 0.048] | 0.961 | 0.952 | 0.053 |
| Friends’ perceived social norms | 0.059 | [0.022, 0.098] | 0.979 | 0.958 | 0.026 |
| Attitudes towards homosexuality | 0.069 | [0.061, 0.077] | 0.862 | 0.840 | 0.051 |
| Attitudes towards trans women | 0.081 | [0.071, 0.092] | 0.926 | 0.909 | 0.039 |
| Attitudes towards migrants | 0.071 | [0.037, 0.109] | 0.963 | 0.927 | 0.019 |

*Note.* Fit indices resulting from the confirmatory factor analyses.

### 3 Figure 1. Attitudes towards Homosexuality: Moderation Analysis

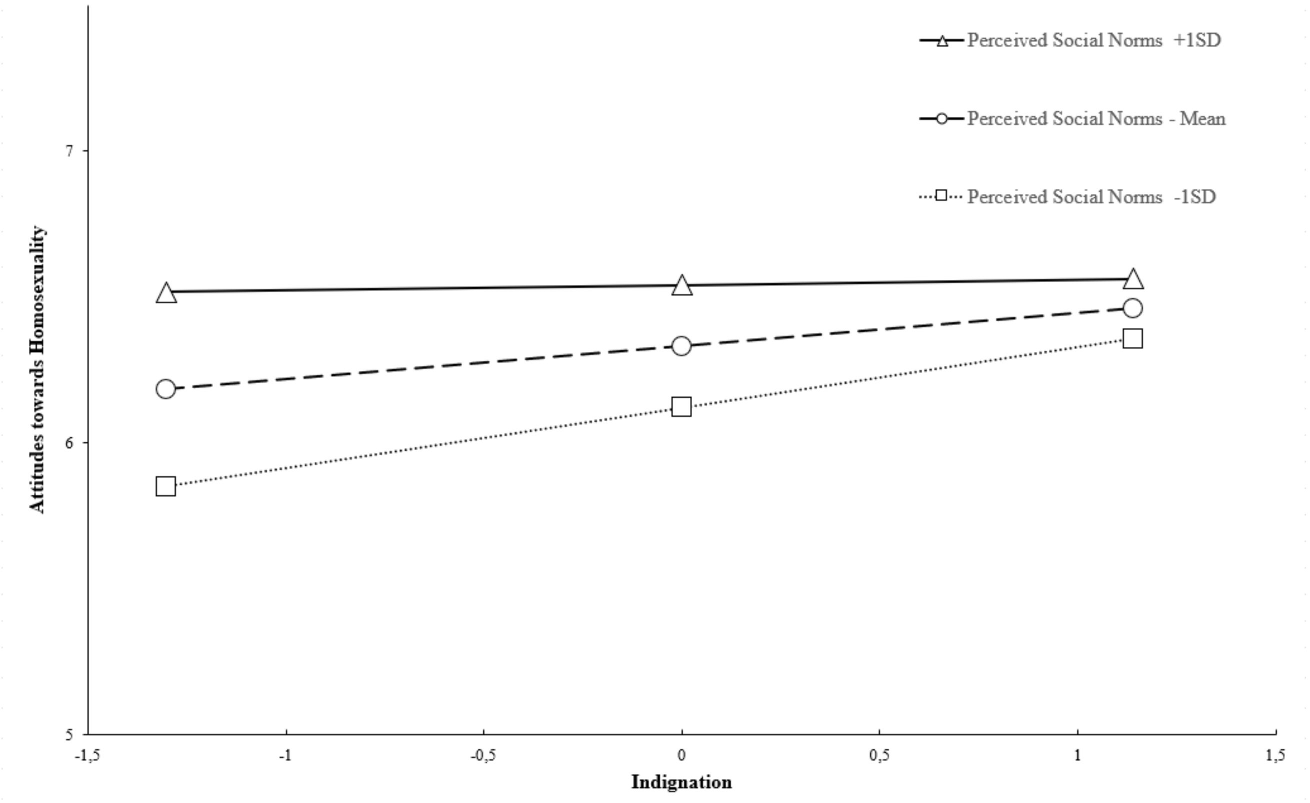


*Note.* Graphical representation of the moderation effect of perceived social norms on the relationship between indignation and attitudes towards homosexuality.

**4 Figure 2. Attitudes towards Trans Women: Moderation Analysis**

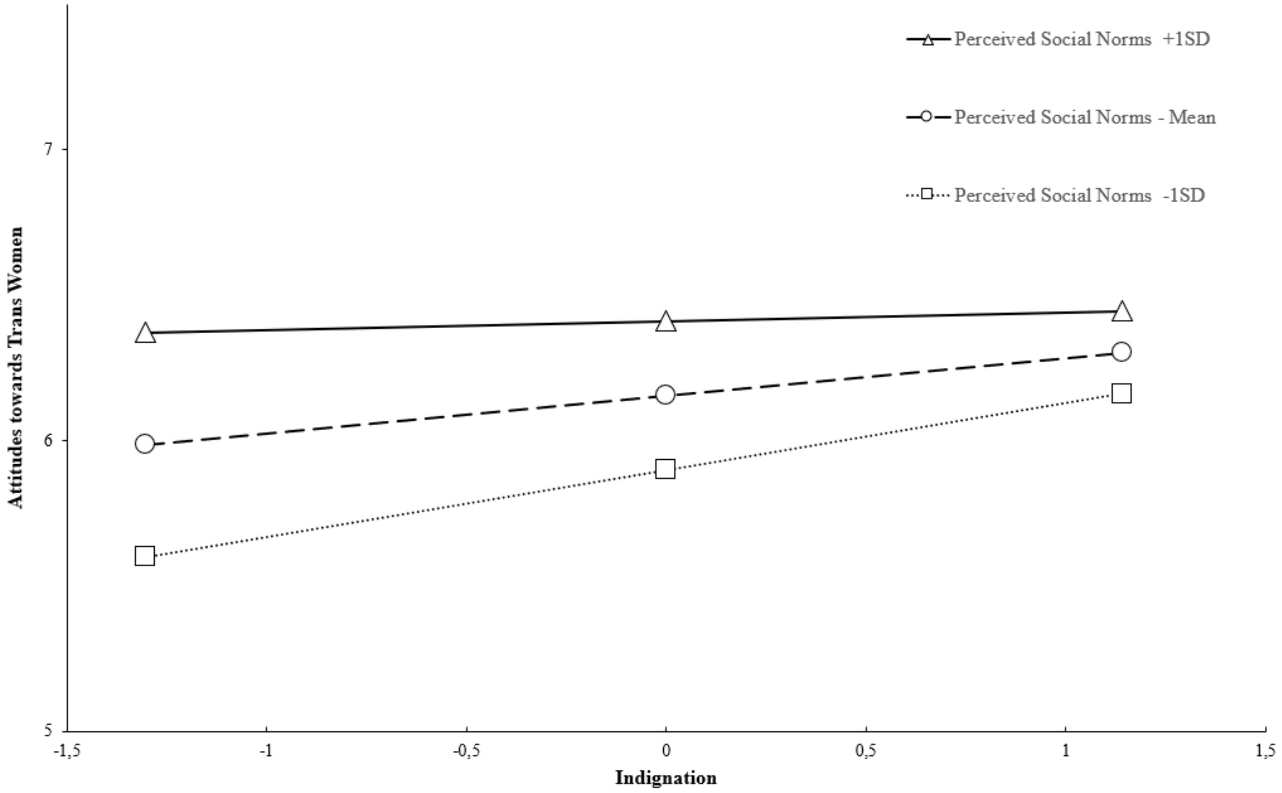


*Note.* Graphical representation of the moderation effect of perceived social norms on the relationship between indignation and attitudes towards trans women.
